# The Relationship Between Weight‐Adjusted Waist Index (WWI) and Cognitive Function in Older Adults: A Cross‐Sectional Analysis of the NHANES 2011‐2014 Data

**DOI:** 10.1002/alz.095696

**Published:** 2025-01-09

**Authors:** Xichenhui Qiu, Changning Wei

**Affiliations:** ^1^ Shenzhen University, Shenzhen, Guangdong China; ^2^ New York University, New York, NY USA; ^3^ Shenzhen Polytechnic University, Shenzhen China

## Abstract

**Background:**

The impact of obesity on cognitive function has engendered considerable interest. Weight‐adjusted waist index (WWI) emerged as a novel and innovative marker of obesity that has responded to weight‐independent abdominal obesity. However, the association between WWI and cognitive function remains unclear. To address this gap, the present study aims to explore the relationship between weight‐adjusted waist index (WWI) and cognitive performance in older adults.

**Method:**

The datasets from the National Health and Nutrition Examination Survey (NHANES) 2011‐2014 were used in a cross‐sectional investigation. Multivariate regression and smoothing curve fitting were used to investigate the linear and nonlinear relationship between WWI and cognitive function, which was tested by neuropsychological tests, including CERAD‐WL, AFT, and DSST, respectively. Subgroup analysis and interaction tests were used to test whether this relationship was stable across groups. Machine learning models based on random forest facilitated the analysis of associations between WWI and cognitive metrics.

**Result:**

A total of 3472 participants were involved in the analysis. Results showed that WWI was significantly negatively associated with low scores on the CERAD‐WL [‐0.96 (‐1.30, ‐0.62)], AFT [‐0.77 (‐1.05, ‐0.49)], and DSST [‐3.67 (‐4.55, ‐2.79)], and this relationship remained stable after converting the WWI to a categorical variable. In addition, this significant negative association was more pronounced in men than women and diminished with advancing age. Non‐linear threshold effects were noted, with the correlations intensifying between WWI and CERAD‐WL when WWI surpassed 12.25, AFT when WWI surpassed 11.54, and DSST when WWI surpassed 11.66.

**Conclusion:**

A higher WWI, indicative of increased abdominal obesity, was correlated with deficits in learning, memory, verbal fluency, and processing speed among older adults. The results suggest that abdominal obesity may be an essential factor in cognitive decline in older adults. The results demonstrated the importance of interventions to reduce abdominal obesity in protecting cognitive performance in older adults.

